# Comparative study on the efficacy of non-steroidal, steroid and non-use of anti-inflammatory in the treatment of acute epidemic conjunctivitis ^1^

**DOI:** 10.1590/s0102-865020190120000006

**Published:** 2020-02-07

**Authors:** Luiz Alfredo Santiago, Jussara Matyelle Rodrigues da Silva, Orleâncio Gomes Ripardo de Azevedo, Paulo Roberto Leitão de Vasconcelos

**Affiliations:** I Master in Surgery, and Physician, Hospital de Olhos Leiria de Andrade, Fortaleza-CE, Brazil. Acquisition of data, technical procedures, manuscript writing.; II Fellow Master degree, Pharmacology Department, Universidade Federal do Ceará (UFC), Fortaleza-CE, Brazil. Statistical analysis, analysis and interpretation of data, technical procedures.; III Pos Doc, Postgraduate Program, Department of Surgery, UFC, Fortaleza-CE, Brazil. Manuscript preparation and writing, critical revision; IV PhD, Full Professor, Coordinator, Postgraduate Program in Surgery, Department of Surgery, UFC, Fortaleza-CE, Brazil. Intellectual and scientific content of the study, critical revision, final approval.

**Keywords:** Conjunctivitis, Adrenal Cortex Hormones, Cytokines

## Abstract

**Purpose:**

To evaluate the effects of prednisolone against sodium diclofenac both with ciprofloxacin compared to artificial tears on the symptoms and signs of acute viral conjunctivitis.

**Methods:**

Study included 37 patients diagnosed with acute conjunctivitis and distributed by three groups: A (1% prednisolone acetate + ciprofloxacin (0.3%); B (Sodium diclofenac (0.1%) + ciprofloxacin (0.3%) and C (artificial tears + ciprofloxacin (0.3%). Patients received medication 6/6 hours daily. Signs and symptoms (e.g. lacrimation, burning, photophobia, etc.) were scored at baseline and on the first, third, fifth and seventh days and in the end of treatment using a standardized questionnaire and slit lamp anterior segment examination.

**Results:**

All three groups demonstrated an improvement in the signs and symptoms of conjunctivitis in their follow-up visits. There was no significant difference in symptom and sign scores between Group A and B and B and C in the study visits ( *p* >0.05). However, the comparison between groups A and C showed a clinical trend (p=0.05) on third evaluation suggesting better clinical action using the corticosteroids.

**Conclusion:**

The prednisolone acetate was not superior to the use of sodium diclofenac or artificial tears in relieving the signs and symptoms of viral conjunctivitis.

## Introduction

Approximately 70% of patients with acute conjunctivitis present to their primary care provider or an urgent care center rather than to an ophthalmologist^1^ . Conjunctivitis is a common complaint in primary care, affecting all ages and socioeconomic classes affecting 6 million people annually in the United States^2 , 3^ .

Infectious conjunctivitis can have several etiologic factors, such as bacterial, viral, chlamydial, fungal, and parasitic. In addition, non-infectious conjunctivitis includes allergens, toxicities, and irritants^4^ .

Common viral agents include adenovirus, herpes simplex, herpes zoster, and enterovirus. Allergic conjunctivitis encompasses seasonal allergic conjunctivitis, perennial allergic conjunctivitis, vernal keratoconjunctivitis (VKC), atopic keratoconjunctivitis (AKC), and giant papillary conjunctivitis^4^ .

Conjunctivitis can be further divided into acute or chronic types. Acute conjunctivitis is characterized by onset within 3 to 4 weeks of the presentation and chronic is defined as more than 4 weeks in duration^5^ .

Acute conjunctivitis is usually a self-limiting condition; however, it is important to rule out other sight-threatening red eye diseases. Viral conjunctivitis is an extremely common cause of conjunctivitis, with estimates as high as 80% of all causes of acute conjunctivitis^6^ .

As many as 90% of these cases of viral conjunctivitis are thought to be caused by human adenovirus, which is known to cause 2 distinct syndromes: epidemic keratoconjunctivitis and pharyngoconjunctival fever^7^ .

Viral conjunctivitis is highly contagious^1 ,5^. The virus spreads through direct contact via contaminated fingers, medical instruments, swimming pool water, or personal items; in one study, 46% of infected people had positive cultures grown from swabs of their hands^8^ . Because of the high rates of transmission, hand washing, strict instrument disinfection, and isolation of the infected patients from the rest of the clinic has been advocated^9^ . Incubation and communicability are estimated to be 5 to 12 days and 10 to 14 days, respectively^10^ .

Although no effective treatment exists, artificial tears, topical antihistamines, or cold compresses may be useful in alleviating some of the symptoms^11^ . Available antiviral medications are not useful^10^ and topical antibiotics are not indicated^11^ .

In hospitalized patients, a randomized study compared topical ketorolac 0.5% and indomethacin 0.1% to artificial tears^12^ . Ketorolac and indomethacin were more effective in decreasing conjunctival hyperemia, but burning, foreign--body sensations, and photophobia were unaffected. In a different randomized study of 117 patients, topical ketorolac 0.5% used four times daily was no better than artificial tears in relieving signs (conjunctival injection, chemosis, mucus, and lid edema) and symptoms (itching, foreign body sensation, tearing, redness, lid swelling, and overall discomfort) of viral conjunctivitis^13^ .

In a rabbit ocular model to evaluate antiviral activity, neither 0.5% ketorolac nor 0.1% diclofenac demonstrated inhibitory activity on viral replication or the formation of subepithelial immune infiltrates. In contrast, 1% prednisolone acetate prolonged viral shedding. Thus, the objective of this study was to evaluate the effects of the administration of prednisolone against sodium diclofenac both associated with ciprofloxacin compared to artificial tears on the symptoms and signs of acute viral conjunctivitis.

## Methods

The research project, with the experimental protocol and the consent term, was submitted to the Research Ethics Committee, Universidade Federal do Ceará, accredited by CONEP – Conselho Nacional de Saúde / MS and approved - Protocol No. 118/11 according with the Helsinki Declaration of 1975 as revised in 2008. All the protocols were adequate to the resolution 466/12 of Health Ministry.

In the current study, 37 patients, 20 women and 17 men, were divided into 3 groups; A (n=16), B (n=5) and C (n=16), constituting people of both genders with ages between 18 and 70 years. Patients were invited based on clinical history and slit lamp examination, where they were examined prior to initiation of treatment and at the end of the study, being followed up every 3 days.

To guarantee the homogeneity of the characteristics between the study groups, a block randomization was adopted^11^ . Fixed-size blocks of nine envelopes were used, of which 3 were with medication group A, 3 with group B and 3 with group C. At the beginning of each block a lottery was made to indicate which medication to use.

The medication was administered for 15 days of treatment with corticoid eye drops (1% prednisolone acetate) and sodium diclofenac sodium eye drops. Antibiotic eye drops (ciprofloxacin 0.3%) were also used, because it is common for epidemic conjunctivitis of viral cause, to complicate with a bacterial infection ^12^ . For the relief of symptoms in group C, artificial tears were used.

### Symptoms assessment

For overall assessment of relief, patients answered the following question: Do you consider the relief of conjunctivitis symptoms satisfactory during the last 15 days? The answer was yes or no.

### Inflammatory markers panel

Dosage of inflammatory mediators: through conjunctival smears and conjunctival secretion, with an absorbent ophthalmic sponge, performed only in the most symptomatic eye^13 , 14^ , at the last evaluation. The kit - cat HCYTOMAG - 60K - 07 was used to measure the following cytokines: IFNg, IL – 1a, IL - 6, IL - 8, IL - 10, IL - 13, TNF-a. Kit 46-702 MAG / MILLIPLEX MAP^Ⓡ^, for NFkB. Kit cat # MBS723617 for iNOS.

After collection the sponge was placed in the 1.5ml Eppendorf^Ⓡ^ tube, where they were centrifuged 5 minutes at 8000rpm. With forceps the sponge was disposed. For analysis it was ideal to obtain at least 40 microliters. Samples were stored in a drum with liquid nitrogen.

### Statistics

The Friedman test was applied in each group and in total. When the independent variable had three or more groups ANOVA was used with Bonferri posttest for multiple comparisons. The P<0.05 was considered to characterize the statistical difference between the groups.

## Results

This report analyzed 37 patients, 54.1% (20/37) female and 45.9% (17/37) male. The study divided the subjects in three groups A, B and C, all the patients did not demonstrate any significant difference, in the beginning of the experiment, between the median of symptoms 36.8 (±1.9), 29.8 (±2.8), and 32.1(±2.2) respectively ( Table 1 ).

**Table 1:** Comparison of means of symptoms by group and period, with 95% CI.

Evaluations	A	B	C	Total
Pre-study	36.8 (±1.9)	29.8 (±2.8)	32.1 (±2.2)	33.8 (±1.4)
1st evaluation	26.2 (±1.8)	26.3 (±2.0)	25.1 (±1.9)	25.7 (±1.1)
2nd evaluation	11.9 (±1.8)	15.7 (±4.0)	17.9 (±2.4)	15.0 (±1.4)
3rd evaluation	5.8 (±1.1)	14.1 (±3.8)	13.4 (±1.9)	10.2 (±1.2)
4th evaluation	1.1 (±0.5)	10.2 (±3.6)	5.3 (±1.3)	4.1 (±0.9)
Closing	0.0 (±0.0)	6.7 (±2.4)	1.1 (±0.4)	1.4 (±0.5)
P-value*	<0.001	<0.001	<0.001	<0.001

The table shows the means of symptoms along the points of evaluation during the treatment demonstrating a decreasing of the symptoms despite of the protocol of treatment.

The values correspond to the following nomenclature: mean (± 95% CI).

*Friedman test.

Source: Data generated by the author.

All patients demonstrated a decrease in the symptom severity during the period of evaluation. However, group A demonstrated an important decrease of 54.5% from the 1^st^ to 2^nd^ evaluation, while groups B and C showed a decrease of 40.3% and 28.9% respectively. Group A (n=16) received prednisolone (1%) plus ciprofloxacin (0.3%) demonstrated a trend in improvement of the symptoms when compared to group C (n=16) that received artificial tears plus ciprofloxacin (0.3%) in third evaluation (p=0.055); however, no statistical difference was observed between A and B or between B and C groups in the 1^st^, 2^nd^ 3^rd^, 4^th^ evaluation and in the last assessment respectively ( Table 2 ).

**Table 2:** Comparison of means of symptoms between groups, by period, with 95% CI.

Period of evaluation	A	B	C	P-value		

				A -- B	A -- C	B -- C
Pre-study	18.8 (±1.9)	15.2 (±2.8)	16.4 (±2.2)	0.981	>0.999	>0.999
1st evaluation	13.4 (±1.8)	13.4 (±2.0)	12.8 (±1.9)	>0.999	>0.999	>0.999
2nd evaluation	6.1 (±1.8)	8.0 (±4.0)	9.1 (±2.4)	>0.999	0.996	>0.999
3rd evaluation	2.9 (±1.1)	7.2 (±3.8)	6.8 (±1.9)	0.944	0.055	>0.999
4th evaluation	0.6 (±0.5)	5.2 (±3.6)	2.7 (±1.3)	0.538	0.307	>0.999
Closing	0.0 (±0.00)	3.40 (±2.45)	0.6 (±0.4)	0.301	0.757	0.857

Source: Data generated by the author.

The values correspond to the following nomenclature: mean (± 95% CI).

*Friedman test.

Collected tears were assessed for inflammation using an inflammatory marker panel as described in methods. There were no statistical differences observed between the groups studied, IFNg (p=0.447), IL-10 (0.505), IL-13 (0.641), IL-1a (0.859); IL-8 (0.177); TNF-a (0.735) ( Table 3 ).

**Table 3:** Levels of inflammatory markers in groups A, B and C.

Mediator	Median	IC 95%	Group A		Group B		Group C		P-value

			Median	IC 95%	Median	IC 95%	Median	IC 95%	
IFNy	4.58	(1.91 – 11.05)	2.80	(1.10 – 11.05)	28.37	(1.39 – 467.00)	8.01	(2.94 – 19.15)	0.447
IL-10	8.67	(2.64 – 27.06)	5.31	(1.45 – 23.97)	206.43	(1.33 – 775.00)	23.29	(5.54 – 53.34)	0.505
IL-13	17.07	(11.88 – 26.67)	18.42	(2.79 – 30.22)	12.38	(12.38 – 19.81)	25.50	(8.54 – 43.45)	0.641
IL-1a	11.70	(8.55 – 21.59)	11.79	(4.24 – 43.81)	57.28	(0.45 – 133.00)	11.70	(8.11 – 22.58)	0.859
IL-6	20.03	(13.52 – 35.62)	9.46	(5.97 – 22.10)	74.81	(6.24 – 618.00)	26.22	(17.66 – 70.19)	0.184
IL-8	441.00	(254.00 – 681.00)	445.00	(215.00 – 681.00)	1366.50	(254.00 – 2927.00)	254.00	(94.61 – 803.00)	0.177
TNFa	10.98	(5.63 – 26.36)	12.79	(2.71 – 37.81)	69.28	(0.68 – 928.00)	8.81	(5.63 – 28.12)	0.735
iNOS	0.00	(0.00 – 0.00)	0.00	(0.00 – 0.00)	0.00	(0.00 – 0.00)	0.00	(0.00 – 0.00)	-

Source: Data generated by the author.

The values correspond to the following nomenclature: mean (± 95% CI).

*Friedman test.

## Discussion

Report from the Wills Eye Hospital at Florida demonstrated prevalence of 62% of adenoviral conjunctivitis amongst all subjects presenting clinical diagnosis of infectious conjunctivitis^14 , 15^ . Viruses are associated with up to 80% of all prevalence of acute conjunctivitis^16 - 20^ .

65 to 90% of cases of viral conjunctivitis are caused by adenoviruses, and it produces two common clinical symptoms related to viral conjunctivitis: pharyngoconjunctival fever (high fever, pharyngitis and bilateral conjunctivitis) and keratoconjunctivitis (lymphadenopathy). In another report from Southeastern Brazil, in Sao Paulo, the authors found a prevalence of 59.0% with acute conjunctivitis associated with adenovirus diagnosed by PCR^21^ . In addition, in Northeastern Brazil, in Fortaleza, a study assessing 24 patients demonstrated that 12 (50%) tested positively for viral infection^22^ .

This report did not show any significant differences between treatment with NSAID and steroids in signs of conjunctivitis. Keratoconjunctivitis is frequently associated with outbreaks, and is also commonly related to the adenovirus serotypes^25 , 26^ .

The treatment of keratoconjunctivitis targets reduction in redness, itching, tearing, blurry vision, chemosis and eyelid oedema^14 , 26^ . To this end, there are a variety of topical preparations, each working on a different phase in the inflammatory process, in order to manage those signs^28^ . The treatment of conjunctivitis includes steroids^23^ and NSAID^24^ . This report aimed to demonstrate that the use of anti-inflammatory NSAIDs or corticosteroid could improve the inflammatory status in eye mucosa during conjunctivitis. However, the study could not demonstrate a statistical difference between the treatments. Nevertheless, the report found a clinical trend in the third evaluation between the groups A compared to group B.

In another similar report evaluating sixty patients clinically diagnosed with a different kind of conjunctivitis [Seasonal Allergic Conjunctivitis (SAC)], the patients were treated with diclofenac (0.1%) and ketorolac (0.5%), and the authors demonstrated an improvement on the symptoms (e.g., burning/stinging, discharge / tearing, photophobia, foreign body sensation and swollen eye) in both treatments. In final analysis the therapeutic response did show an improvement on incomes in group treated with ketorolac (0.5%)^28^ . On the other hand, in a study from Recife, Brazil, the authors, evaluating fifty patients with symptoms of acute viral conjunctivitis, a group of twenty-four patients treated with ketorolac (0.45%) plus carboximetilcelulose and the other group with twenty-six patients received artificial tears, demonstrated no statistical difference between the two types of treatment^29^ .

Nevertheless Swany *et al* .^30^ , analyzing eight clinical studies with 712 patients involved with allergic conjunctivitis, demonstrated that use of NSAID produced significant relief of inflammatory conjunctival itching; however, for the other inflammatory signs (e.g. ocular burning/pain, eyelid swelling, photophobia and foreign sensation), the data were not significant, similar to the findings of this study.

Thus, based on this report there is limited evidence to support the use of topical NSAID in viral acute conjunctivitis. Further studies are needed to compare the efficacy of non-hormonal anti-inflammatory, hormonal (steroid) and non-use of anti-inflammatory in acute epidemic and endemic conjunctivitis, through dosage of inflammatory mediators, and clinical status using a higher number of patients.

In this report, no equivalent work was found, being considered the first research in this specific aim. Our observation suggests that in the current study, there was a clinical difference in the group treated with corticoid; however, it was not statistically significant.

## Conclusion

There was no statistical difference between the treatments tested, but in the clinical symptoms of the patients, we can report that there was a clinical trend of improvement for those who received corticosteroid.

## Footnotes

1 Research performed at Hospital de Olhos Leiria de Andrade, Fortaleza-CE, Brazil.
